# High-Frequency Glacial Lake Mapping Using Time Series of Sentinel-1A/1B SAR Imagery: An Assessment for the Southeastern Tibetan Plateau

**DOI:** 10.3390/ijerph17031072

**Published:** 2020-02-08

**Authors:** Meimei Zhang, Fang Chen, Bangsen Tian, Dong Liang, Aqiang Yang

**Affiliations:** 1 Key Laboratory of Digital Earth Science, Institute of Remote Sensing and Digital Earth, Chinese Academy of Sciences, No. 9 Dengzhuang South Road, Beijing 100094, China; zhangmm@radi.ac.cn (M.Z.); tianbs@radi.ac.cn (B.T.); liangdong@radi.ac.cn (D.L.); yangaq@radi.ac.cn (A.Y.); 2 University of Chinese Academy of Sciences, Beijing 100049, China; 3 Hainan Key Laboratory of Earth Observation, Institute of Remote Sensing and Digital Earth, Chinese Academy of Sciences, Sanya 572029, China

**Keywords:** glacial lake mapping, Tibetan Plateau, median composite, Sentinel-1, time series

## Abstract

Glacial lakes are an important component of the cryosphere in the Tibetan Plateau. In response to climate warming, they threaten the downstream lives, ecological environment, and public infrastructures through outburst floods within a short time. Although most of the efforts have been made toward extracting glacial lake outlines and detect their changes with remotely sensed images, the temporal frequency and spatial resolution of glacial lake datasets are generally not fine enough to reflect the detailed processes of glacial lake dynamics, especially for potentially dangerous glacial lakes with high-frequency variability. By using full time-series Sentinel-1A/1B imagery over a year, this study presents a new systematic method to extract the glacial lake outlines that have a fast variability in the southeastern Tibetan Plateau with a time interval of six days. Our approach was based on a level-set segmentation, combined with a median pixel composition of synthetic aperture radar (SAR) backscattering coefficients stacked as a regularization term, to robustly estimate the lake extent across the observed time range. The mapping results were validated against manually digitized lake outlines derived from Gaofen-2 panchromatic multi-spectral (GF-2 PMS) imagery, with an overall accuracy and kappa coefficient of 96.54% and 0.95, respectively. In comparison with results from classical supervised support vector machine (SVM) and unsupervised Iterative Self-Organizing Data Analysis Technique Algorithm (ISODATA) methods, the proposed method proved to be much more robust and effective at detecting glacial lakes with irregular boundaries that have similar backscattering as the surroundings. This study also demonstrated the feasibility of time-series Sentinel-1A/1B SAR data in the continuous monitoring of glacial lake outline dynamics.

## 1. Introduction

Glacial lakes are important indicators of the regional glacier dynamics in response to climate warming and changing precipitation [1,2,3]. With the continuous retreating and thinning of mountain glaciers in the Tibetan Plateau, a large number of glacial lakes have developed and experienced rapid expansion in recent decades, leading to the increased hazard risk of glacial lake outburst floods (GLOFs) [4]. These GLOFs usually evolve and erupt over a short time and can trace long distances from high altitude regions, which will pose a great threat to the life and property in the downstream valleys [5,6].

Knowledge about the glacial lake distribution and their development are of great importance for the better understanding of the associated glacial processes and hazard assessment of GLOFs [7,8]. Multitemporal mapping of glacial lakes is the first step in the evaluation of the potential hazard from GLOFs since it can be used to perform the analysis of the evolution patterns of glacial lakes in order to identify the potentially dangerous glacial lakes on the regional scale [9,10]. Terrestrial surveying has provided the traditional tools for studying glacial lakes for several decades. However, one of the great limitations of these techniques is that they have difficulties in accessing generally remote glacial lakes over a large and risky mountainous terrain area, and they are extremely costly in terms of time and money. In contrast, remote sensing techniques can provide an archive of satellite images obtained both from optical and microwave sensors over a long time, and are the only feasible tools for the detailed investigation of glacial lakes without the great expense of field measurements [11].

Optical remote sensing data, such as Landsat series satellite data, Sentinel-2A/2B, Advanced Spaceborne Thermal Emission and Reflection Radiometer (ASTER), Systeme Probatoire d’Observation de la Terre-5 (SPOT-5), and Moderate-resolution Imaging Spectroradiometer (MODIS) data, have been widely applied for studies related to glacial lake dynamic monitoring and hazard management [12,13,14,15]. Based on these data, visual interpretation that depends on subjective empirical judgment is the main method used for the delineation of mountainous glacial lakes characterized by complex terrain conditions and glaciated environments [11,16,17]. Many studies have usually used the threshold segmentation of normalized difference water indices (NDWIs) for classifying glacial lakes [12,18,19]. Recently, a systematic approach that integrates the advantages of the threshold segmentation method and the Chan-Vese (C-V) model was proposed, which can effectively extract glacial lakes by removing the noise from the surroundings, mountain shadows, and adjacent glaciers [20]. For the accurate extraction of glacial lakes from heterogeneous backgrounds, an automated scheme that uses the non-local active contour approach based on the modified NDWI has been used to produce highly reliable glacial lake maps across the entire Tibetan Plateau region [21]. Although good results have been achieved through these techniques, the data acquisition is strongly hampered by weather conditions and the time of day. Selection of high-quality optical images for lake mapping over large glaciated areas is not an easy task. Furthermore, most of these studies have tried to monitor the glacial lakes by only covering a few time intervals. Because glacial lakes that are prone to outburst are likely to experience dramatic changes over several days, high-frequency mapping offers the valuable information required for the early identification of related hazards.

In this paper, the southeastern Tibetan Plateau was chosen as the test site because glacial lakes in this region are more sensitive to climate change, abundant precipitation, and high ice-layer temperatures, and show high intra-annual variability in the lake extent. Moreover, due to interference from the cloudy and rainy climate, the limited data availability made the exploration of glacial lake distribution and evolution patterns even more challenging. Synthetic aperture radar (SAR) data has the advantages of all-weather and full-time capabilities. This study utilizes the free availability and short revisit cycle (six days) of Sentinel-1A/1B SAR data, and aimed to develop an effective, automated, and high-temporal-resolution methodology for extracting the glacial lake outlines. We combined the full time series of the Sentinel-1A/1B data over the southeastern Tibetan Plateau using a pixel-compositing approach involving derived backscattering coefficient stacks, examined medians to robustly estimate the glacial lake extent for each date using an improved level-set method, and demonstrated the ability of this median-based compositing approach to deal effectively with data quality, abnormal values, and other issues that commonly affect single-scene methodologies.

## 2. Study Area and Data

### 2.1. Study Area

**Figure 1:**
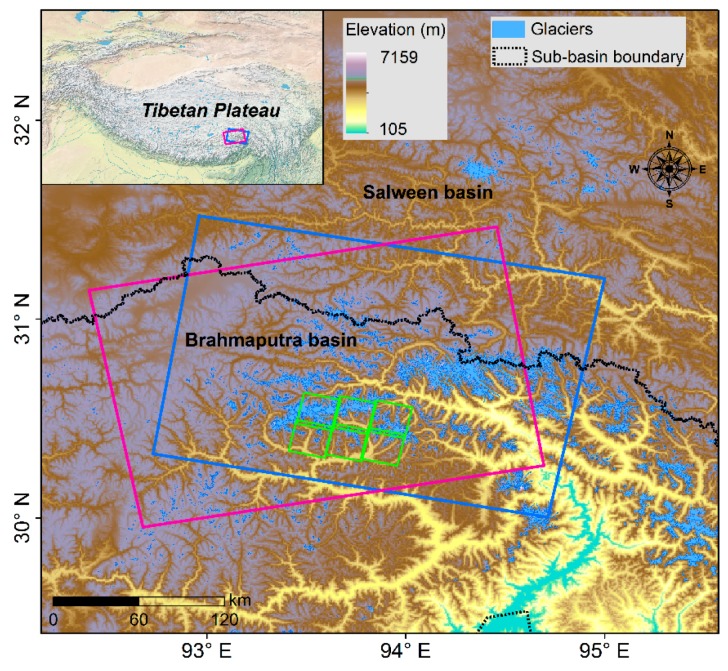
Geographic location of study area with the topographical characteristics and distribution of mountain glaciers in the two river basins. Pink and blue rectangles represent the frames of ascending and descending Sentinel-1 images, respectively. Green rectangles indicate the coverage of Gaofen-2 panchromatic multi-spectral (GF-2 PMS) images used for accuracy evaluation of the glacial lake mapping results.

Our study area was located in the southeastern Tibetan Plateau, which includes parts of two sub-basins: Salween Basin and Brahmaputra Basin (corresponding to the area of one ascending and one descending Sentinel-1A/1B frame; see Figure 1). The climate of this region is mainly influenced by two atmospheric circulation systems [22]. The dry season period is from November to April, with little precipitation and cold climate provided by the southern branch of the mid-latitude westerlies [23]. May to October is the wet season; during this time, the westerlies become weak and the Indian monsoons penetrate the region, indicating the beginning of rainy season. The heavy rainfall in the wet season accounts for 60–90% of the annual total [24]. The terrain and geomorphology of southeastern Tibet are rather complex and diverse, and form a landscape of alpine canyons. Monsoonal temperate glaciers have developed over a large range across the region. These monsoonal temperate glaciers are more sensitive to climate warming and abundant precipitation, and are thinning and melting faster than continental glaciers. Affected by the combined interactions from local climate conditions and higher ice-layer temperatures, glacial lakes in this region are much larger and deeper, and generally shown as navy blue in color [9,20].

### 2.2. Data

In this study, according to the mapping and validation aims, three different types of images in different time periods were selected. Table 1 gives a summary of all the data used. For the development of the classification method and mapping of the high-frequency glacial lakes, all available time series of C-band Sentinel-1A/1B ground range detected (GRD) images from both the ascending and descending orbits at a spatial resolution of 10 m, were accessed from Google Earth Engine cloud computing platform (https://code.earthengine.google.com/) with a consecutive time interval of six days. These data were mainly acquired during May to October 2018 in order to ensure that most of the glacial lakes were not frozen and were easily visible in the images [18], and to further ensure the reliability and representative of the median composite pixel values derived from these intensity images. This median-based compositing approach can deal effectively with data quality, abnormal values, and other issues that will affect single scene classification. Among the mapping results, some glacial lakes at a particular time were used for the accuracy assessment, which were mapped based on the time series of the C-band Sentinel-1A/1B images from the ascending and descending orbits in July to August 2018 to be consistent with the time period of the validation data from Gaofen-2 (GF-2) panchromatic multi-spectral (PMS) imagery. For the validation of the lake mapping results, we used six cloud-free GF-2 images acquired in July to August 2018 from the descending orbits to evaluate the accuracy of the mapping results of glacial lakes in a similar time period. Here, the GF-2 scenes were chosen due to the fact that they had a high spatial resolution of 1 m/4 m in the panchromatic/multi-spectral bands and a revisit cycle of five days, which is quite similar to the revisit period of the dual satellite constellation of Sentinel-1A and Sentinel-1B. The basically consistent acquisition dates for the Sentinel-1A/1B and GF-2 images can reduce the influence of lake extent fluctuations on the two-date lake maps comparison.

Furthermore, Digital Elevation Model (DEM) data were obtained to serve as the topographic reference for the geocoding of SAR and optical data, and for removing the mountain shadows that may induce the classification errors for lake mapping. This study used the ASTER Global Elevation Model Version2 (GDEM V2) gridded data with a 30-m resolution collected from http://gdem.ersdac.jspacesystems.or.jp/.

## 3. Methods

**Figure 2:**
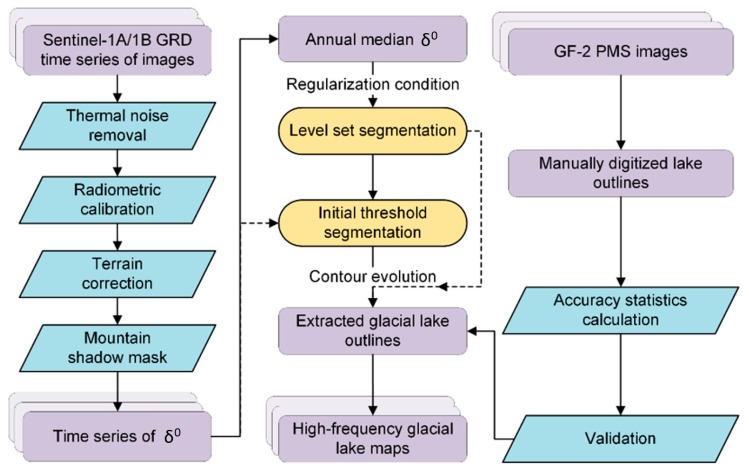
Flowchart of our method for mapping the glacial lakes at a high temporal resolution based on the time series of Sentinel-1A/1B SAR data.

Figure 2 shows the detailed mapping procedure of our proposed method for glacial lakes. We first gathered time-series Sentinel-1A/1B images and pre-processed them for water identification. Then, based on the median composite of the backscattering coefficients, an improved segmentation method was developed for extracting the glacial lake outlines from a single date. Finally, the obtained Sentinel-based glacial lake outlines were validated with the manual digitization of lake boundaries from GF-2 PMS images. We describe each process in more detail in the following subsections.

### 3.1. Pre-Processing of Images

Each Sentinel scene was pre-processed with the Sentinel-1 Toolbox using the following steps: (1) thermal noise removal, which mainly removes additive noise [25]; (2) radiometric calibration, which refers to computing backscatter intensity values using sensor calibration parameters; (3) terrain correction using ASTER GDEM V2, where the final terrain-corrected values are converted to decibels; and (4) mountain shadow mask, where because potential lake areas are generally on a flat surface, a slope larger than 15° was set to remove the mountain shadows [20].

To assess the accuracy of the lake extraction results from the SAR data, the GF-2 imagery was geocoded on a Universal Transverse Mercator map projection. Because the spatial resolution of the multispectral image was not that high for use in the precise evaluation of lake maps, the reference lake outlines were manually delineated on the panchromatic image.

### 3.2. Extracting the Time Series of the Glacial Lake Outlines

**Figure 3:**
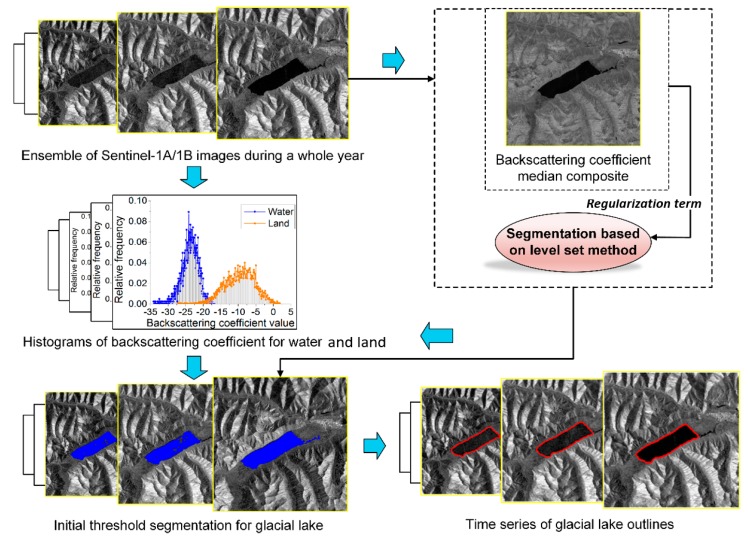
Conceptual graph of the basic steps in the process of mapping time series of glacial lakes.

For each Sentinel observation, the backscattering coefficient at each pixel was calculated, which created a stack of backscattering coefficient images. Individually, due to outliers and other artefacts, each backscattering intensity image could be used to roughly estimate the glacial lake area. This could be performed using a threshold based on an image histogram for water and land (Figure 3). As a result, the initial extraction results for glacial lakes for each date were generated.

To effectively deal with the noise factors in a single image, we performed the pixel–based compositing method, which created the pixel values that were most representative of the full dataset and were unaffected by the outliers or noise in each data [26]. In this study, in order to estimate the representative value in a simple and well-understood way, we took the median value at each pixel. Using the median helped to avoid a lot of potential problems with the SAR data observations, such as the presence of speckle noise that may affect individual intensity values, residual terrain shadows not completely removed by slope measurements, and shallow water or outflow drainage occasionally occurring at a particular time that may result in misclassification [3,27].

During the implementation of the mapping method [28,29], the median composite of the backscattering coefficient was added as a regularized term to improve the computational efficiency. The initial outline of the glacial lake was used as a good initial contour, as it was usually very close to the real lake shape, saving more time for the processing. Finally, all the glacial lake outlines were progressively and automatically updated for each acquisition date. This method was fast and effective with a low labor intensity due to the rough location of a given lake and the regularization term of the median values.

### 3.3. Validating the Glacial Lake Mapping Results

By comparing with the glacial lake outlines that were manually digitized from the GF-2 images, the Sentinel-based glacial lake maps were assessed. Due to the variable nature of some glacial lake conditions and the surrounding environment, the difference in the image acquisition date between Sentinel-1A/1B and GF-2 images were no more than five days in order to ensure the reliability of the evaluation results. Commission and omission errors were calculated for all lake and land pixels together. A low percentage error in the water and land classes suggests an accurate classification. Moreover, the kappa coefficient and overall accuracy were also computed from these errors. In addition to the above detailed accuracy analyses, further validation of the robustness of the proposed method was undertaken by comparing the glacial lake maps generated using support vector machine (SVM) and Iterative Self-Organizing Data Analysis Technique Algorithm (ISODATA) classification methods.

## 4. Results and Discussion

### 4.1. Accuracy Assessment

**Figure 4:**
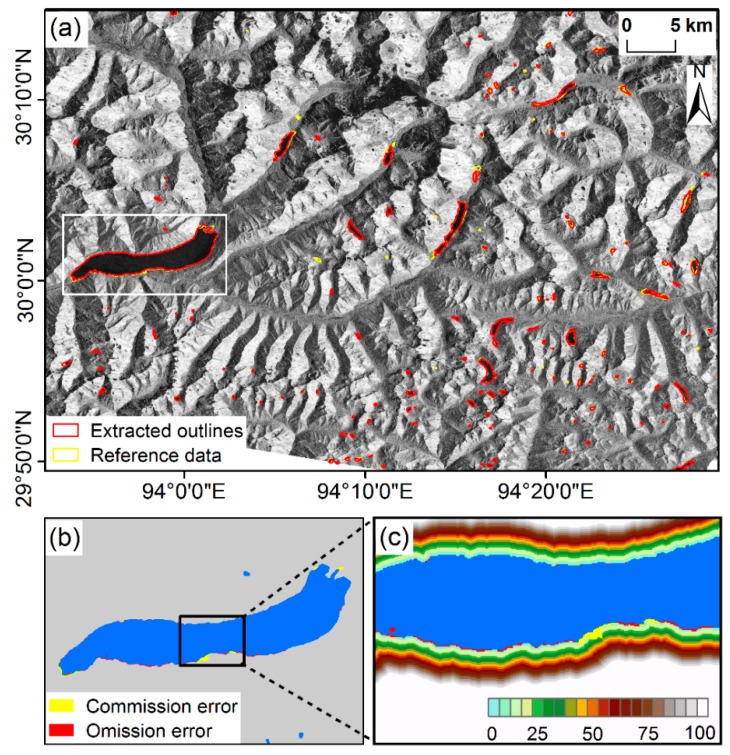
Extraction results for some test glacial lakes. (**a**) Comparison of manually digitized glacial lake outlines from GF-2 PMS images and extracted outlines from Sentinel-1A/1B images using the proposed method. (**b**) Commission errors (indicated as yellow color pixels) and omission errors (indicated as red color pixels) of the typical glacial lake. (**c**) Enlarged distance buffer zone (in pixels) image around the outline for the glacial lake in (b).

The validation was conducted on a total of 283 glacial lakes that were covered by the area of GF-2 imagery. As this study area was large and glacial lakes were abundant at a small size, it was difficult to show all the lake boundaries and classification errors clearly. For a better display and visualization of the extraction results and their classification accuracy, Figure 4 presents a complete example of the accuracy assessment that was performed on the mapping results of some test glacial lakes. As shown in Figure 4a, the extracted glacial lake outlines from the Sentinel-1A/1B image (red contours) were almost coincident with the manually digitized glacial lake outlines (yellow contours); these clearly illustrate that the proposed method worked well for detecting glacial lakes from the SAR data. To show more details about the accuracy assessment results, a relatively large glacial lake was selected as the example shown in Figure 4b. From Figure 4b, it can be seen that the misclassification mainly occurred along the lake outline, and the number of commission and omission pixels were low. This can be further confirmed using the enlarged distance buffer zone around the outline of this glacial lake (Figure 4c), where the distance between the extracted and manually digitized glacial lake outline was less than five pixels.

**Figure 5:**
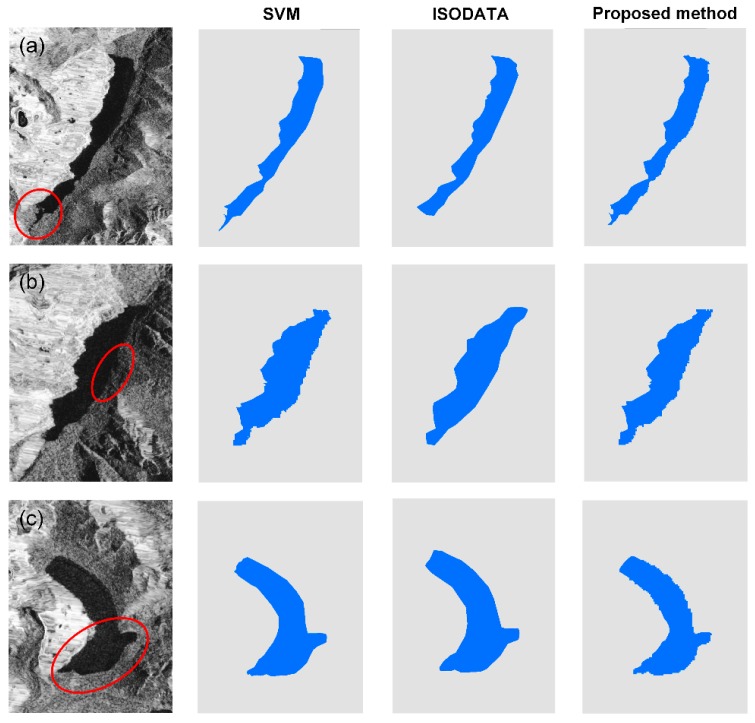
Comparison of the glacial lake extent extracted from Sentinel-1A/1B data in 2018 using SVM, ISODATA, and the proposed method for three sampled glacial lakes at: (**a**) 94.25°E, 30.05°N; (**b**) 93.64°E, 30.24°N; and (**c**) 94.28°E, 29.91°N.

To show the advantages of the proposed method, we also utilized the supervised SVM and unsupervised ISODATA methods for comparison. The comparison was also conducted for the glacial lakes in the area covered by the GF-2 PMS images. Figure 5 illustrates the differences between the glacial lake outlines extracted by the three methods. Here, three glacial lakes that were characterized by a complex geometrical shape and various surroundings were selected as typical examples to show the detailed comparative results. For the glacial lake (a) that had an irregular shape and stream outlets in the lower reaches of the lake (indicated as a red ellipse in Figure 5a), the SVM overestimated this water area but it was completely ignored by the ISODATA, reflecting their poor ability in the delineation of glacial lakes with complex curved shapes [30]. However, the proposed method took the median composite of the backscattering coefficient values as the regularization term to restrain the lake boundary and could accurately extract the water bodies in the lower reaches region of the glacial lake. For some glacial lakes that had a similar backscattering with the background, as is shown in the eastern part of the glacial lake (b) (Figure 5b), this mainly referred to the local low areas and mud patches characterized by a high moisture content, such that there would still be a tendency to overestimate the glacial lake area using SVM from a single image. The ISODATA method extracted a rough water boundary, which was because it applied global statistics to generate a clustered center, which is often hindered in highly complex images, especially for complex water conditions [31,32]. On the contrary, the proposed method exhibited the best performance due to its superiority in terms of spectral and backscattering variability. The last glacial lake (c) could be accurately identified using all three methods (Figure 5c), but our method outperformed the SVM and ISODATA since it could capture more local details and obtain a sharper border along the edge of the lake.

We also quantitatively evaluated the classification accuracy for these 283 glacial lakes in the area covered by the GF-2 PMS images, and Table 2 summarizes the statistical accuracy results. Generally, the higher omission errors relative to the commission errors demonstrated that the missing glacial lake pixels were the main source of classification errors for all three methods. The highest accuracy, with a kappa coefficient of 0.95 and overall accuracy of 96.54%, was achieved for the classification results obtained using the proposed method. The commission and omission errors for the water and land classes were also very low at no more than 4%. The lowest kappa coefficient and overall accuracy were for the map produced using the ISODATA method (kappa = 0.79, overall accuracy = 88.87%). Better results were achieved for the SVM classification method, which needed a lot of user expertise and proper training samples, and had a kappa coefficient and overall accuracy of 0.85 and 92.13%, respectively. These quantitative evaluation results show that the proposed median-based composite method was effective and accurate enough to extract glacial lakes from the SAR intensity data.

### 4.2. Temporal Variation of Glacial Lakes during the Year of 2018

**Figure 6:**
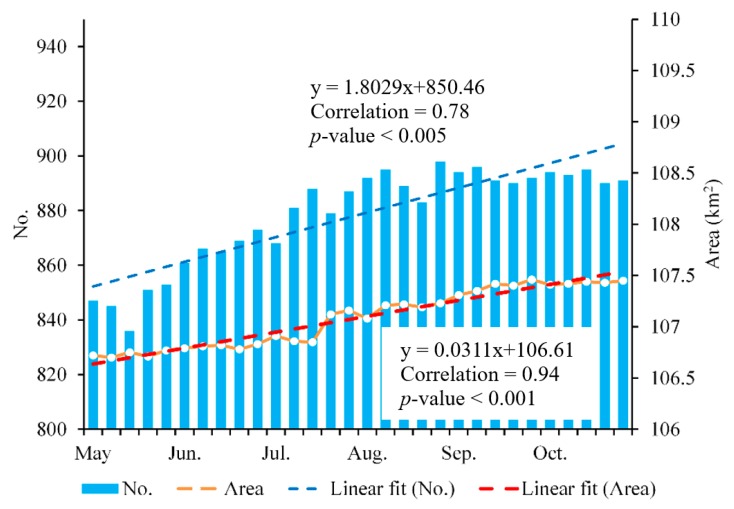
Seasonal changes in the number and area of glacial lakes from May to October 2018.

In the high mountainous areas, some glacial lakes develop rapidly, i.e., within some weeks or a few months [33,34]; therefore, low-frequency monitoring is not sufficient to track changes and detect hazardous developments in a timely manner. In these conditions, high-frequency monitoring would be highly valuable for capturing the seasonal changing pattern of glacial lakes and for the early detection of related hazards. Here, we examine the applicability of the developed method for the continuous monitoring of glacial lakes in the study area. Figure 6 shows the multi-temporal glacial lake change detection from Sentinel-1A/1B SAR data using the proposed method. In the whole study region of the southeastern Tibetan Plateau, there were 847 glacial lakes in early May, covering a total area of 106.72 km^2^. The lake number showed an increasing trend during this period, with an average increase of about two lakes for each revisit cycle of six days. Meanwhile, the total area of the glacial lakes also increased with time, increasing by 0.73 km^2^ until October (107.45 km^2^), which was a +0.68% change relative to May. A linear fit to all the data shows a mean expansion rate of 0.031 km^2^ per six days. Based on the analysis of temporal changes, a relatively high seasonal variability of glacial lakes was detected in this study, which revealed the formation and expansion of glacial lakes between May and October.

### 4.3. Glacial Lake Mapping Results in the Study Area

**Figure 7:**
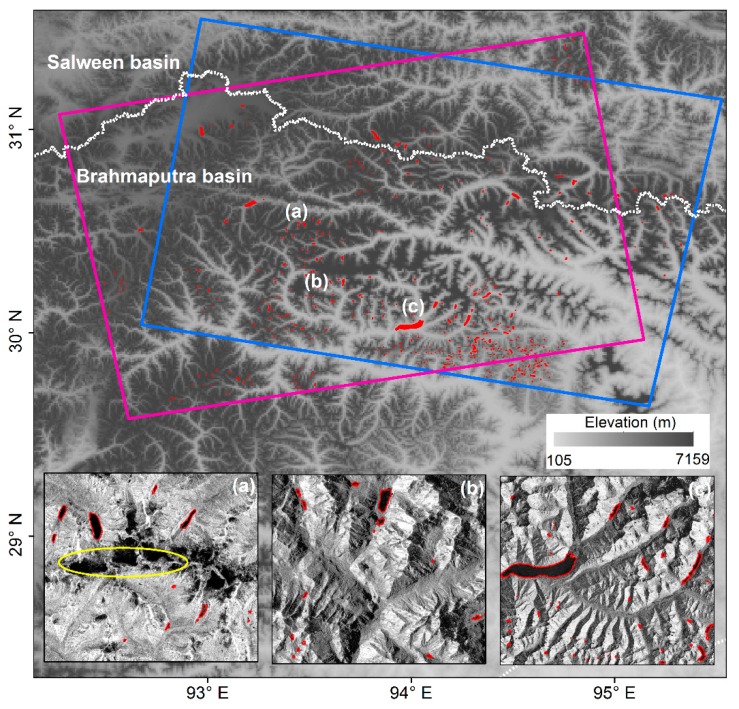
The spatial distribution of glacial lakes (shown with red) extracted using the developed method over the southeastern Tibetan Plateau (indicated as pink and blue rectangles). Three magnified maps (**a**–**c**) in the bottom show the local details in the lake outlines overlaid on the Sentinel-1A/1B median composite image.

In order to test the performance of the Sentinel-1A/1B data with the developed median-composite based segmentation method over large areas, experiments covering the whole study region of the southeastern Tibetan Plateau were carried out, as shown in Figure 7. Three sub-regions that were characterized by different environmental conditions were selected as typical examples for more local details. The glacial lakes were formed in the junction area of the Salween and Brahmaputra Basins, and were more densely distributed in the Brahmaputra Basin. Almost all the glacial lakes in this region could be categorized into an unconnected glacial lake type, namely lakes that were not in directly contact with current glaciers, due to the accelerating retreat of glaciers induced by the warm and humid climate. Some of these glacial lakes were impounded by block or debris dams [35], exhibiting a clear boundary in the SAR intensity image. However, their confusion with the terrain shadow areas (e.g., yellow ellipse in Figure 7a) must be considered because of the very low backscattering intensity that is similar to that of water. In this study, terrain shadows are effectively resolved using a threshold applied on slopes derived from DEM and the meaningful median estimator for the intensity values. Other lakes, including a large number of small glacial lakes, were embedded in undulating landscapes or impounded by rock barriers [36], filling with water due to both abundant rainfall and glacier meltwater (Figure 7b,c). These results suggest that the combination of the time series of Sentinel-1A/1B SAR imagery and the median composite mapping method would provide reliable and accurate information of the temporal evolution of glacial lakes over the large and rugged alpine areas, and allow for the identification of potentially dangerous glacial lakes.

### 4.4. Discussion

In many glacial lake mapping methods, the lack of high-frequency mapping results is a problem for some hazardous lakes that have high seasonal variations, making it difficult for timely and reliable GLOF risk evaluation. The use of a glacial lake inventory dataset with a coarse temporal resolution may lead to the incorrect analysis of the evolution patterns of glacial lakes, which may also affect decision-making regarding hazard management. This study devised a method that was devoted to increasing the frequency maps of glacial lakes by utilizing the time series of Sentinel-1A/1B SAR data with consecutive 6-day periods. In addition to the high-frequency characteristics, our method also showed an improvement in accuracy when compared with SVM and ISODATA methods in classifying complex-shaped glacial lakes with various surroundings. It should be noted that in our study, to reduce the influence of data noise and uncertainty from a single image, all the accessible images acquired during the observation period were used to compute the median value for each pixel for the precise delimitation of the lake extent. In classifying images that did not have a sufficient enough image number or high data quality, the derived median values may differ slightly and affect the extraction results of glacial lakes from what is mapped in this study.

As for the selection of features for segmentation, in this paper, we used the SAR intensity images to extract the glacial lake outlines, which have extensive applications for water delineation due to its low backscattering values at all microwave wavelengths and distinct separation from the snow, glacier, and debris cover around the glacial lakes [27,37]. This allows for the fairly accurate classification of glacial lakes in intensity images. Given the fact that the relative instability of glacial lake water will significantly decrease the degree of interferometric synthetic aperture radar (InSAR) coherence, the capability of coherence has been recently explored to characterize and map the unconnected glacial lakes in the Himalayas [37], which has proved to be a promising feature for deriving lake boundaries and may be used as an input feature for future study. Furthermore, the application of the proposed method is not limited to SAR data since it can be extended for the extraction of glacial lake outlines by incorporating spectral features from optical images, such as single-band reflectance features, water indices, image transformation features, etc.

Nevertheless, in our test cases, we did not consider the influence of different types of glacial lakes, such as proglacial lakes and supra-glacial lakes. Therefore, the robustness of the new method needs to be tested in different regions under various climatic and cryospheric backgrounds. More sites over the whole Tibetan Plateau may need to be included for a thorough evaluation of the performance of the method.

## 5. Conclusions

The high-frequency continuous monitoring of glacial lake dynamics is crucial for the understanding of their distribution, evolution, and the driving mechanism of rapid expansions. However, we currently have limited information of glacial lake dynamics at high frequency due to the low availability of data and lack of advanced techniques. Using time series of Sentinel-1A/1B SAR intensity data with a six-day interval, the new method introduced in this paper contributes toward improving the accuracy of glacial lake mapping and change detection at a high temporal resolution for various environmental studies and applications. This method uses a simple and systematic median composite technique to find the backscattering coefficient for enhancing the water separability, and meanwhile, to remove part of the shadow and dark surface noises, which are often the major causes of misclassification in the mapping of glacial lakes. The median composite values were used as the regularization term in a level-set function to smooth the segmentation boundaries and prevent the occurrence of small, isolated regions in the classification of an individual image. The manually digitized glacial lake outlines based on the GF-2 PMS imagery served as reference data to validate the glacial lake mapping results. Comparing with the SVM and ISODATA classification approach, the new method was shown to extract glacial lakes with the highest overall accuracy of 96.54%, particularly in mountainous areas where there were complex terrain conditions. This method was also shown to be effective for large glaciated areas in the southeastern Tibetan Plateau.

## Figures and Tables

**Table 1:** List of Sentinel-1A/1B images for the method development and glacial lake mapping experiment, and corresponding GF-2 PMS images used for validation.

Purpose	Platform and Sensor	Pass Direction	Time Period or Date (dd/mm/yyyy)
Segmentation method development and high-frequency glacial lake mapping	Sentinel-1A/1B	Ascending/Descending	May to October, 2018
Some glacial lake mapping results used for accuracy assessment	Sentinel-1A/1B	Ascending	3 July, 2018
		Ascending	15 July, 2018
		Descending	21 July, 2018
		Descending	2 August, 2018
		Ascending	8 August, 2018
		Ascending	20 August, 2018
Validation	GF-2 PMS	Descending	7 July, 2018
		Descending	12 July, 2018
		Descending	22 July, 2018
		Descending	1 August, 2018
		Descending	6 August, 2018
		Descending	16 August, 2018

**Table 2:** Summary of classification accuracy of three different methods.

Method	Class	Commission Error (%)	Omission Error (%)	Kappa	Overall Accuracy (%)
SVM	Water	4.86	6.89	0.85	92.13
	Land	5.65	6.94		
ISODATA	Water	7.62	8.95	0.79	88.87
	Land	8.49	9.14		
Proposed method	Water	1.03	2.72	0.95	96.54
	Land	0.38	3.91		
